# Hyperglycemia-triggered ATF6-CHOP pathway aggravates acute inflammatory liver injury by β-catenin signaling

**DOI:** 10.1038/s41420-022-00910-z

**Published:** 2022-03-14

**Authors:** Chao Yang, Zeng Wang, Yuanchang Hu, Shikun Yang, Feng Cheng, Jianhua Rao, Xuehao Wang

**Affiliations:** Hepatobiliary Center, The First Affiliated Hospital of Nanjing Medical University; Key Laboratory of Liver Transplantation, Chinese Academy of Medical Sciences, 210029 Nanjing, Jiangsu Province China

**Keywords:** Acute inflammation, Stress signalling

## Abstract

Although hyperglycemia has been documented as an unfavorable element that can further induce liver ischemia–reperfusion injury (IRI), the related molecular mechanisms remain to be clearly elaborated. This study investigated the effective manner of endoplasmic reticulum (ER) stress signaling in hyperglycemia-exacerbated liver IRI. Here we demonstrated that in the liver tissues and Kupffer cells (KCs) of DM patients and STZ-induced hyperglycemic mice, the ER stress-ATF6-CHOP signaling pathway is activated. TLR4-mediated pro-inflammatory activation was greatly attenuated by the addition of 4-phenylbutyrate (PBA), one common ER stress inhibitor. The liver IRI in hyperglycemic mice was also significantly reduced after PBA treatment. In addition, deficiency of CHOP (CHOP^−/−^) obviously alleviates the hepatic IRI, and pro-inflammatory effects deteriorated by hyperglycemia. In hyperglycemic mice, β-catenin expression was suppressed while the ATF6-CHOP signal was activated. In the liver tissues of PBA-treated or CHOP^−/−^ hyperglycemic mice, the expression of β-catenin was restored. Furthermore, CHOP deficiency can induce protection against hyperglycemia-related liver IRI, which was disrupted by the knockdown of β-catenin will cause this protection to disappear. High glucose (HG) treatment stimulated ATF6-CHOP signaling, reduced cellular β-catenin accumulation, and promoted the TLR4-related inflammation of BMDMs. But the above effects were partially rescued in BMDMs with CHOP deficiency or by PBA treatment. In BMDMs cultured in HG conditions, the anti-inflammatory functions of CHOP^−/−^ were destroyed by the knockdown of β-catenin. Finally, chimeric mice carrying WT or CHOP^−/−^ BMDMs by bone marrow transplantation were adopted to verify the above conclusion. The current study suggested that hyperglycemia could trigger ER stress-ATF6-CHOP axis, inhibit β-catenin activation, accelerate inflammation, and deteriorate liver IRI, thus providing the treatment potential for management of sterile liver inflammation in DM patients.

## Introduction

Metabolic diseases including diabetes, obesity, and metabolic syndrome have become chronic diseases that are prevalent worldwide. According to the International Diabetes Federation, >9.3% of diabetes mellitus (DM) cases in 2014 (96,290,000 cases) occurred in China. DM is one major type of metabolic disease characterized by chronic hyperglycemia caused by disorders of insulin secretion and/or utilization caused by multiple etiologies. The persistent hyperglycemia in patients will further induce a variety of diabetic complications, such as cardiovascular and cerebrovascular complications [1–4]. Diabetes is also associated with poor prognosis related to ischemia–reperfusion injury (IRI). A series of clinical reports indicate that the kidney, nervous, heart, and liver tissues of diabetic patients tend to have more severe IRI [5–8].

IRI is one of the main causes of liver dysfunction after liver transplantation, liver trauma, and liver resection [9–12]. In liver transplantation cases, IRI is not only associated with 10% of early transplant failures but also involved in the occurrence of acute and chronic rejections [12]. Therefore, alleviating IRI during liver transplantation will help increase the success rate and treatment effect of liver transplantation. However, the occurrence and development of liver IRI involve many different factors, and its mechanism remains complicated.

Physiological responses during IRI, such as hypoxia and calcium disorder disrupt the homeostasis of the endoplasmic reticulum (ER), leading to the accumulation of unfolded/misfolded proteins in the ER cavity, and inducing ER stress [13–15]. To restore ER homeostasis, ER stress stimulates unfolded protein response (UPR), including translation inhibition, increased accumulation of ER chaperones and associated proteins, and degradation of unfolded/misfolded proteins [16]. Although UPR can prevent cells from excessive apoptosis [17], continuous UPR and ER stress can still cause inflammation and cell death [18–20].

Diabetes is the main reason for accidental readmissions of patients undergoing liver surgery. Compared with non-diabetic patients, diabetic patients have a higher risk of liver transplant failure [21, 22]. Acute hyperglycemia was previously shown to worsen hepatic IRI by amplifying oxidative stress and the inflammatory response to IR in rats [23]. In addition, activation of the AGE-RAGE signaling pathway in Kupffer cells (KCs) triggers a hyper-inflammatory immune response and exacerbates liver cell damage in diabetic/hyperglycemic patients with liver IR [8]. Activation of β-catenin plays a critical role in maintaining immune homeostasis. Activation of Wnt/β-catenin signaling was shown to promote tissue damage repair via STAT6-dependent M2 polarization [24]. However, the mechanism by which hyperglycemia exacerbates liver IRI has not yet been determined. In addition, whether ER stress-β-catenin signaling is involved in regulating diabetic liver cell damage is still not clear.

In the present study, we demonstrated that the hyperglycemia-triggered ATF6–CHOP axis aggravated IR-induced liver injury via inactivation of β-catenin, which in turn promoted TLR4-driven inflammatory responses, while simultaneously repressing Akt signaling molecules in the ischemic liver.

## Results

### ATF6-CHOP signaling was activated in liver tissues from DM patients and streptozotocin (STZ) mice

**Fig. 1:**
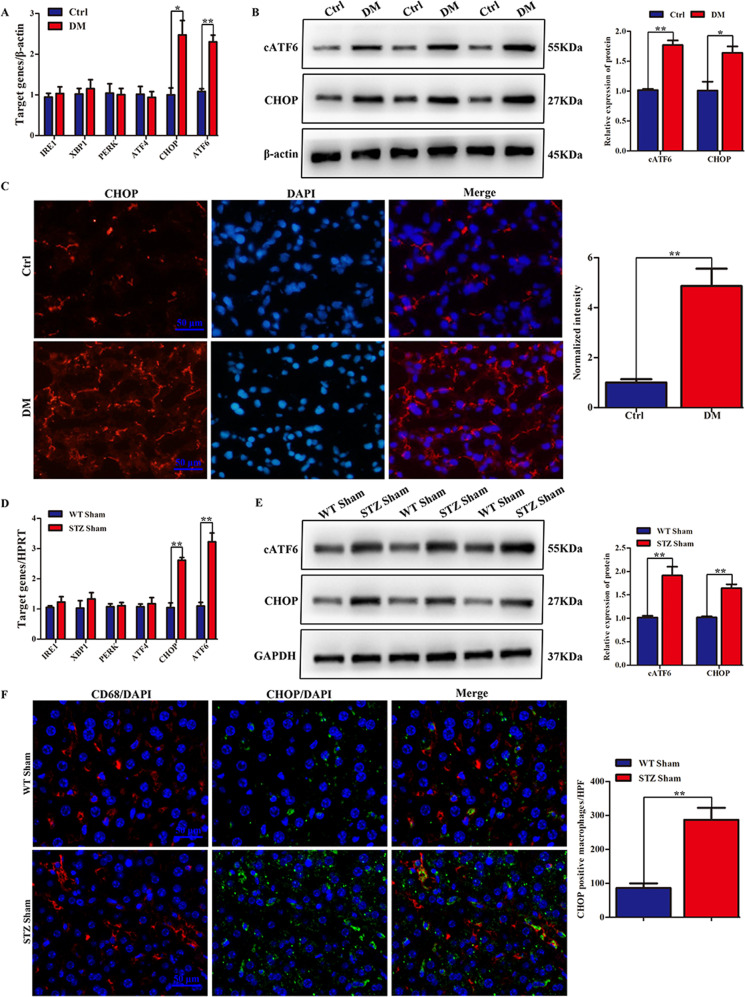
ATF6-CHOP signaling pathway was induced in liver tissues from DM patients and STZ mice. **A** Quantification analysis of mRNA level of ER stress-related pathway molecules (IRE1, XBP1, PERK, ATF4, ATF6, and CHOP) in human livers (*n* = 15). *P < 0.05, **P < 0.01. **B** Protein level detection of cATF6 and CHOP in human liver. **C** CHOP in clinical samples was detected by IF staining. CHOP-positive cells from different groups were counted and presented in the right panel. **P < 0.01. **D** IRE1, XBP1, PERK, ATF4, ATF6, and CHOP gene mRNA levels in mice undergoing sham operation were quantified using qRT-PCR (ratios of target gene/HPRT). (*n* = 4~6). **P < 0.01. **E** The protein expression of cATF6 and CHOP in different groups of mice undergoing sham operation were examined by western blot. **F** Dual-immunofluorescence staining of CD68+ macrophages (red) and CHOP (green) in mouse liver sections. Histograms presented in the right panel were scored semi-quantitatively by averaging the number of positively stained cells per field (×200). Data are based on three independent experiments with similar conclusion. *P < 0.05, **P < 0.01.

To determine whether ER stress was involved in hyperglycemia-exacerbated liver IRI, we analyzed ER-stress-related pathways in liver tissues from DM patients and STZ mice. Human liver specimens were harvested from 15 DM patients and 15 healthy subjects (Table S1). All subjects suffered from benign liver diseases. The level of sALT was significantly elevated in DM patients after postoperative days 1 and 3, but not after 5 days. DM patients had higher expression levels of ATF6 and CHOP, but not IRE1, XBP1, PERK, and ATF4 (Fig. 1A). Consistent with the mRNA expression levels, protein levels of cleaved (c) ATF6 and CHOP were also markedly enhanced in DM patients (Fig. 1B). CHOP, as a downstream molecule of ATF6, was further confirmed to be more highly expressed in DM compared with healthy livers, according to immunofluorescence (Fig. 1C). To determine whether a similar phenomenon occurred in hyperglycemic mice, low-dose STZ was injected into wild-type (WT) C57BL/6 mice. At 2 weeks after STZ injection, whether the mice exhibited a hyperglycemic state was verified (Supporting Fig. S1A), and the livers were harvested. Interestingly, the ATF6-CHOP pathway was activated in a similar manner in STZ-induced hyperglycemic livers (Fig. 1D, E). Then, whether CHOP was activated by hyperglycemia in the macrophage population in vivo was studied. Immunofluorescence (IF) staining exhibited that CHOP and CD68 co-localized in macrophages, and the number of cells expressing CHOP was elevated in STZ-induced hyperglycemic mice (Fig. 1F).

### ATF6-CHOP pathway is critical for hyperglycemia-related liver IRI

**Fig. 2:**
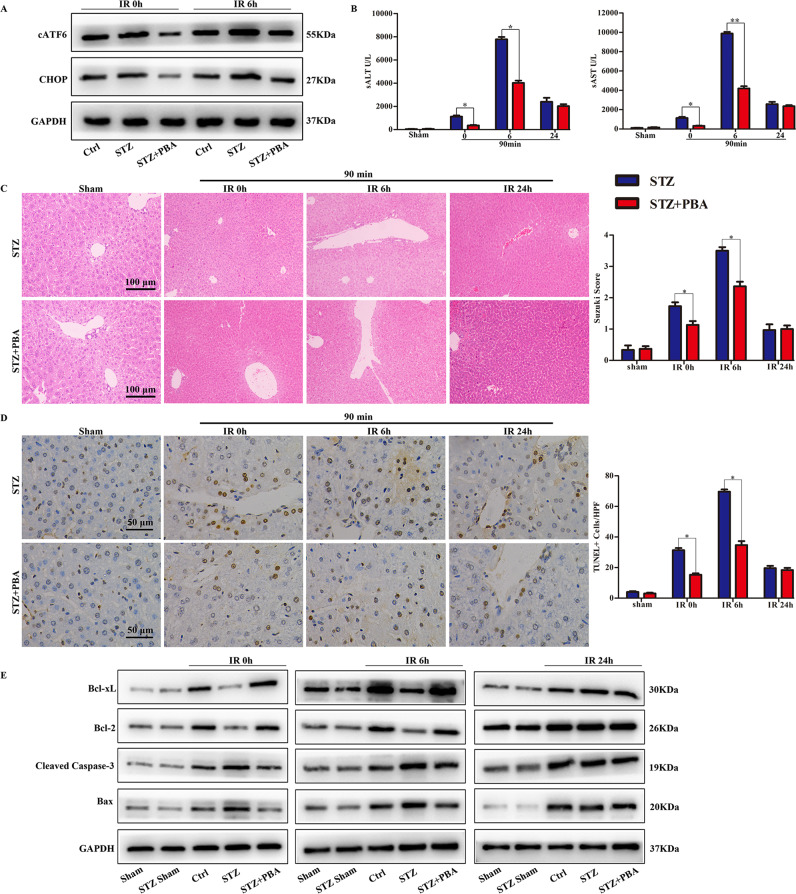
ATF6-CHOP pathway was required for hyperglycemia-exacerbated liver IRI. The ER stress antagonist PBA was administered to DM mice before the start of liver ischemia. Untreated diabetic mice were used as controls. **A** cATF6 and CHOP protein expression in control mice, diabetic mice, and PBA-treated diabetic mice was measured by western blot analysis. Liver IRI was measured at 0, 6, and 24 h by. **B** The quantification results of sALT and sAST. **C** H&E staining of ischemic liver tissue (*n* = 4~6). Suzuki’s histological score was used to evaluate liver damage, and results were presented as a histogram in the right. **D** TUNEL staining of representative ischemic liver lobes (*n* = 4~6). TUNEL+ cells were measured by recording the positive cell numbers per area. **E** Western blot detection of Bcl-xL, Bcl-2, Cleaved caspase-3, Bax, and GAPDH. Data are representative of three experiments. *P < 0.05, **P < 0.01.

To confirm the roles of the hyperglycemia-activated ATF6-CHOP signaling in hepatic IRI, we treated hyperglycemic mice with 4-phenylbutyrate (PBA), western blot results indicated that higher levels of cATF6 and CHOP were detected in the 90 min ischemic livers of hyperglycemic mice at 0 and 6 h of reperfusion, whereas PBA effectively inhibited the hyperglycemia-triggered ATF6-CHOP pathway (Fig. 2A). Liver and serum were harvested at 0, 6, and 24 h after reperfusion. The decreased concentration of sALT and sAST demonstrated that compared with untreated hyperglycemic mice, PBA significantly alleviated liver injury at 0 and 6 h, but not at 24 h after reperfusion (Fig. 2B), PBA also improved liver architecture with lower Suzuki scores (Fig. 2C). Terminal deoxynucleotidyl transferase dUTP nick end labeling (TUNEL) staining showed that PBA reduced the number of TUNEL-positive cells compared with the untreated group at 0 and 6 h after reperfusion (Fig. 2D). The frequency of cleaved caspase-3-positive cells was lower at 0 and 6 h after reperfusion in the PBA-treated group (Supporting Fig. S1B, C). Western blotting assays of Bcl-xL, Bcl-2, cleaved caspase-3, and Bax further supported the idea that PBA treatment effectively inhibited hyperglycemia-enhanced apoptosis in liver IRI (Fig. 2E).

### ATF6-CHOP pathway is essential for hyperglycemia-promoted pro-inflammatory responses in liver IRI

**Fig. 3:**
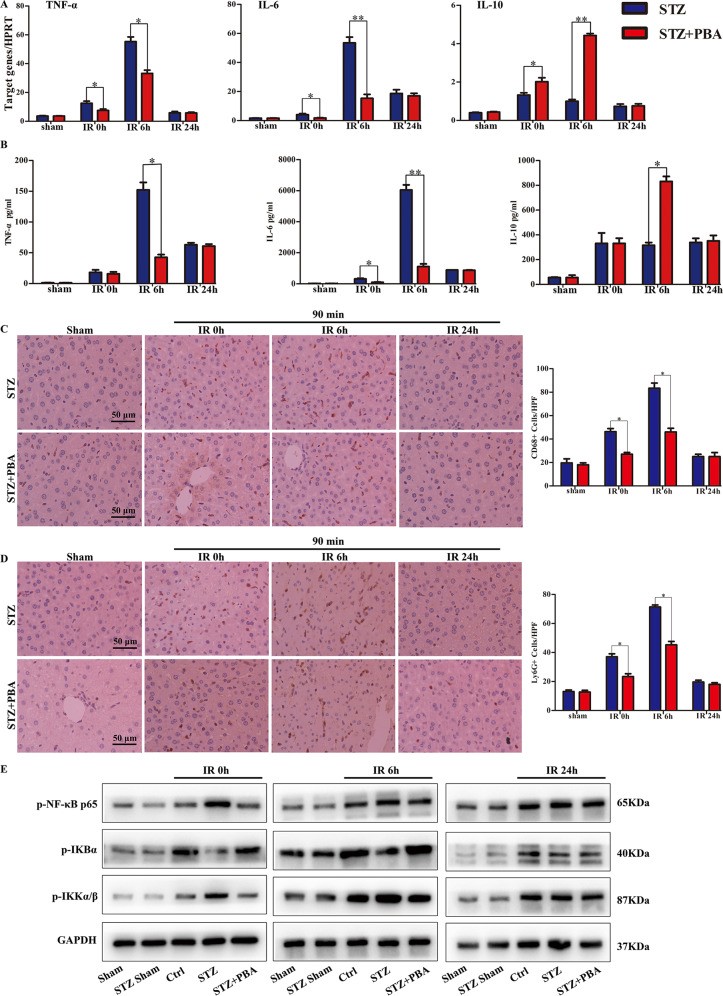
ATF6-CHOP pathway was essential for the hyperglycemia-promoted pro-inflammatory response in liver IRI. **A** TNF-α, IL-6, and IL-10 mRNA levels were examined by qRT-PCR and **B** the serum cytokine levels were measured by ELISA. **C**, **D** The infiltration of neutrophil and macrophage were analyzed by IHC (×200). CD68+ and Ly6G+ cells were quantitated by counting positive cells and the quantified results are shown as the right panels. **E** Protein expression detection of p-IKBα, p-NF-κB p65, p-IKKα/β, and GAPDH. Data are representative of three independent assays with consistent results. *P < 0.05, **P < 0.01.

In order to explore whether the ATF6-CHOP pathway regulates the liver pro-inflammatory response that is exacerbated by hyperglycemia after IR, the levels of tumor necrosis factor (TNF)-α, interleukin (IL)-6 and IL-10 at different time points after untreated and after reperfusion in hyperglycemic mice with or without PBA treatment. PBA notably diminished hyperglycemia-enhanced expression of TNF-α and IL-6, and elevated hyperglycemia-reduced expression of IL-10 at 0 and 6 h after reperfusion (Fig. 3A, B). Regarding macrophage and neutrophil infiltration, there was no significant difference in Ly6G+ neutrophils and CD68+ macrophages in either untreated sham or PBA-treated hyperglycemic sham mice, but compared with untreated hyperglycemic mice, they were notably lower in PBA-treated hyperglycemic mice at 0 and 6 h, but not at 24 h after reperfusion (Fig. 3C, D). To further determine whether PBA treatment reduced hyperglycemia-enhanced inflammatory response by inhibiting the TLR4–nuclear factor (NF)-κB pathway, according to western blot analysis, p-NF-κB p65 and p-IKKα/β activation were extremely higher in hyperglycemic mice compared with control mice at 0 and 6 h after reperfusion, which were reduced in PBA-treated hyperglycemic mice ischemic liver tissues. The opposite effect was observed in relation to p-IKBα protein expression (Fig. 3E).

### CHOP deficiency rescues hyperglycemia-exacerbated liver injury and pro-inflammatory responses after IR

**Fig. 4:**
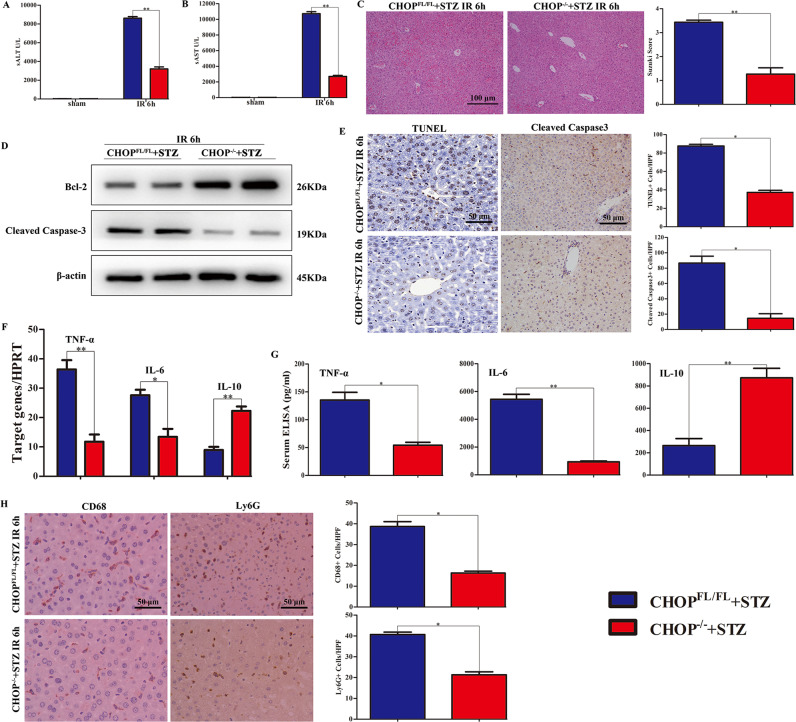
CHOP deficiency alleviated hyperglycemia-exacerbated liver injury and TLR4-mediated innate immune responses after IR. **A**, **B** Liver function was evaluated by the value of sALT and sAST. **C** Histopathologic analysis of livers at 6 h after reperfusion, and the severity of liver IRI was judged according to Suzuki’s histological grading. **D** Protein expression of Bcl-2 and Cleaved caspase-3 in CHOP^−/−^ and WT diabetic mouse ischemic livers was detected using western blot. **E** Apoptosis was evaluated in CHOP^−/−^ and WT diabetic mouse ischemic livers by immunohistological staining with anti-cleaved caspase-3 (×200), and representative TUNEL-stained ischemic liver lobes (magnification ×400). TUNEL+ cells were recorded based on positive cell numbers/area. Cytokines level of liver and serum were examined through qRT-PCR (**F**) and ELISA (**G**), respectively. **H** CD68 and Ly6G staining of ischemic liver lobes (×200). Data are representative of three independent assays with similar results. *P < 0.05, **P < 0.01.

CHOP-deficient (CHOP^−/−^) and WT mice were injected with low-dose STZ, and the liver IRI model was then established. Samples were harvested at 6 h after reperfusion. The level of sALT or sAST in CHOP^−/−^ sham group was similar to the WT sham groups, but CHOP deficiency markedly attenuated hyperglycemia-enhanced sALT and sAST after IR (Fig. 4A, B). These data were in line with Suzuki’s histological grading of liver IRI (Fig. 4C). CHOP deficiency markedly increased hyperglycemia-inhibited Bcl-2 expression and decreased hyperglycemia-enhanced Cleaved caspase-3 expression (Fig. 4D). TUNEL and Cleaved caspase-3 staining also indicated an anti-apoptotic role of CHOP deficiency (Fig. 4E). To determine the inflammatory status, we further examined TNF-α, IL-6, and IL-10 expression in ischemic liver and serum. As expected, CHOP deficiency greatly inhibited the abundance of IL-6 and TNF-α and enhanced IL-10 expression after IR in hyperglycemic mice (Fig. 4F, G). Moreover, we determined whether CHOP disruption affected macrophage and neutrophil trafficking after liver ischemia in hyperglycemic mice. Ly6G+ neutrophils and CD68+ macrophages were both remarkably lower in CHOP^−/−^ than that in WT mice (Fig. 4H).

### Hyperglycemia-triggered ATF6-CHOP pathway depresses β-catenin signaling in liver tissues

**Fig. 5:**
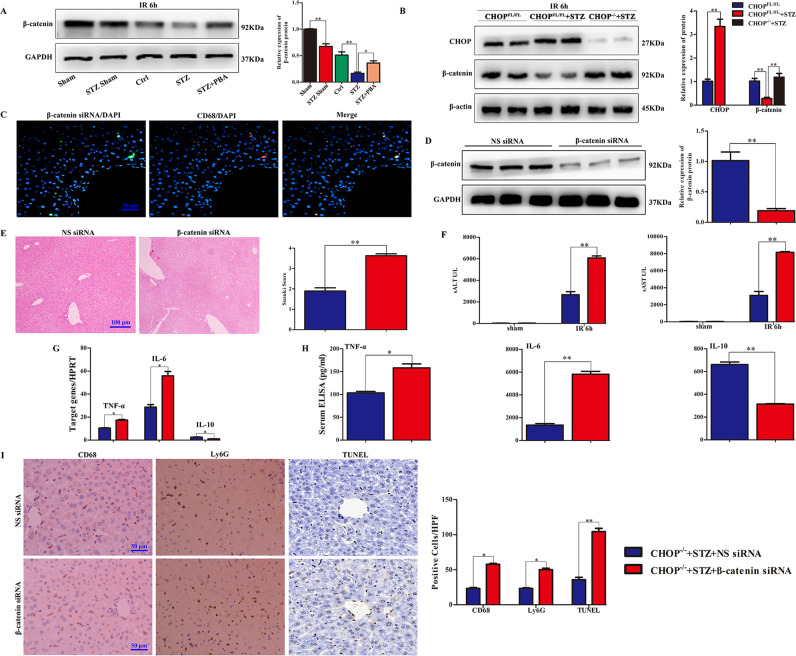
Hyperglycemia-triggered ATF6-CHOP pathway depressed β-catenin signaling in liver tissues, and β-catenin was required for the protective effect of CHOP deficiency in hyperglycemia-aggravated liver IRI. **A** Western blots of β-catenin and GAPDH in sham and ischemic livers, at 6 h post-reperfusion after 90 min ischemia. **B** CHOP and β-catenin protein expression in CHOP^−/−^ and WT diabetic mouse ischemic livers were detected using western blot. CHOP^−/−^ diabetic mice were delivered with Alexa Fluor 488-labeled siRNA through tail vein injection at 4 h before ischemia. **C** IF staining was conducted to exhibit CD68-positive macrophages (red) and control siRNA (green) labeled by Alexa Fluor 488 and in ischemic liver lobes. Nuclei were blue after staining with DAPI. **D** Western blot analysis of β-catenin in KCs isolated from CHOP^−/−^ diabetic mouse administered with NS siRNA or β-catenin siRNA. **E** Histological analysis of ischemic liver tissue. Suzuki’s histological grading was the common standard for judging liver IRI severity. **F** The concentration of sALT and sAST in CHOP^−/−^ DM mice treated with β-catenin siRNA or NS siRNA after 90 min of liver ischemia and 6 h of reperfusion. **G**, **H** The mRNA abundance and protein levels of TNF-α, IL-10, and IL-6 in mouse were determined using qRT-PCR and ELISA, respectively. **I** IHC analysis of CD68+ macrophages and Ly6G+ neutrophils (magnification ×200) and TUNEL staining (magnification ×400) in ischemic livers. *P < 0.05, **P < 0.01. Data are representative of three independent assays.

We analyzed β-catenin expression in hyperglycemic and control mice and showed that hyperglycemia effectively inhibited β-catenin expression in liver tissues compared with the control group (Fig. 5A and Supporting Fig. S2A). In addition, we also determined β-catenin expression in ischemic livers after 6 h reperfusion. Hyperglycemia significantly decreased β-catenin expression, but this was recovered almost to control levels in hyperglycemic mice with PBA treatment (Fig. 5A and Supporting Fig. S2A). To clarify whether hyperglycemia-triggered ATF6-CHOP influenced β-catenin signaling during liver IRI, we examined the signaling in ischemic liver tissues from WT and CHOP^−/−^ hyperglycemic mice after 6 h reperfusion and revealed that CHOP deficiency effectively restored hyperglycemia-inhibited β-catenin expression in control hyperglycemic mice (Fig. 5B and Supporting Fig. S2B).

### β-catenin is required for the protective effect of CHOP deficiency in hyperglycemia-aggravated liver IRI

CHOP deficiency recovered hyperglycemia-inhibited β-catenin expression and alleviated hyperglycemia-enhanced liver pro-inflammatory responses and IRI. We therefore analyzed the role of β-catenin in this process by disrupting β-catenin expression in CHOP-deficient livers via a mannose-linked β-catenin siRNA in vivo. The mannose-linked fluorescence-labeled siRNA was efficiently transduced into macrophages in the liver, whereas hepatocytes were mostly negative for any fluorescent signals (Fig. 5C). After mice were injected with siRNA for 4 h, KCs isolated from CHOP^−/−^ hyperglycemic mouse administered with NS siRNA or β-catenin siRNA post-sham procedure were plated and cultured in vitro. Western blot results confirmed that β-catenin siRNA successfully disrupted the β-catenin expression level in KCs (Fig. 5D). CHOP^−/−^ hyperglycemic mice administered with siRNA-targeting β-catenin had severe liver damage, such as tissue necrosis, edema, sinusoidal congestion, and cytoplasmic vacuolation. Livers in CHOP^−/−^ hyperglycemic mice treated with scramble siRNA presented symptoms of milder liver damage and low levels of necrosis (Fig. 5E). The concentration of sALT and sAST were increased after 6 h reperfusion in the β-catenin siRNA group (Fig. 5F). Moreover, β-catenin siRNA significantly enhanced IL-6 and TNF-α, and down-regulated IL-10 gene transcription in ischemic livers, accompanied by similar trends in serum compared with the NS group (Fig. 5G, H). We stained macrophages and neutrophils in ischemic livers, and showed that β-catenin siRNA administration increased macrophage and neutrophil accumulation compared with the NS siRNA-treated controls, similar trends were observed with respect to apoptosis, by TUNEL staining (Fig. 5I).

### ATF6-CHOP pathway was triggered by HG and promoted pro-inflammatory responses in macrophages

**Fig. 6:**
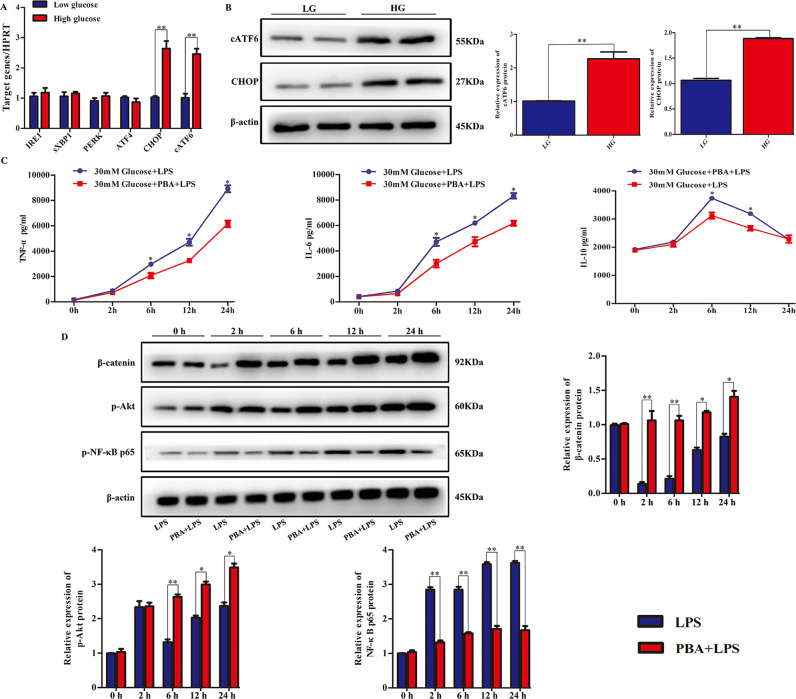
ATF6-CHOP pathway was triggered by HG and promoted pro-inflammatory responses in macrophages. **A** ER stress pathway-related molecules (IRE1, XBP1, PERK, ATF4, ATF6, and CHOP) in BMDMs cultured with LG and HG medium. **B** cATF6 and CHOP protein expression levels measured by western blot. PBA was added into BMDMs cultured in HG DMEM and were stimulated by LPS for another 24 h; untreated BMDMs differentiated under HG conditions were used as controls. **C** Protein levels of TNF-α, IL-6, and IL-10 in culture supernatants measured by ELISA. **D** PBA was added into BMDMs cultured in HG DMEM and were stimulated by LPS for another 24 h. Western blot detection of β-catenin, p-Akt, p-NF-κB p65, and β-actin. *P < 0.05, **P < 0.01. All the results are representative of at least three independent experiments.

Given that hyperglycemia specifically activated the ER stress-ATF6-CHOP pathway and promoted pro-inflammatory responses in hepatic IR injury, we explored whether hyperglycemia had a similar influence on macrophages at the cellular level. Bone marrow-derived macrophages (BMDMs) were differentiated in low glucose (LG) or HG for a week. HG specifically triggered mRNA and protein expression of ATF6 and CHOP, but not IRE1, XBP1, PERK, or ATF4 compared with LG (Fig. 6A, B). To confirm the triggering effects of HG on the ATF6-CHOP pathway in innate immune activation, BMDMs exposed to HG conditions for 7 days were preincubated with PBA for 12 h, and then stimulated with lipopolysaccharide (LPS) for 24 h. BMDMs differentiated under HG conditions produced lower levels of TNF-α and IL-6 (at 6, 12, and 24 h), but higher levels of IL-10 (6 and 12 h) in PBA-treated cells compared with untreated cells (Fig. 6C). To further understand the regulatory mechanisms of the hyperglycemia-triggered ATF6-CHOP pathway in the innate immune response, we detected β-catenin expression in the above-treated BMDMs. β-catenin was rapidly inactivated (at both 2 and 6 h) and then gradually recovered (at both 12 and 24 h), but the β-catenin expression was stabilized by PBA after LPS treatment (Fig. 6D). As shown, PBA markedly enhanced LPS-phosphorylated Akt (Ser473) between 6 h and 24 h while p-NF-κB p65 was decreased in BMDMs at different times under the combination treatment of LPS and PBA (Fig. 6D).

### β-catenin was negatively regulated by CHOP and was critical for HG-mediated inflammatory responses in macrophages

**Fig. 7:**
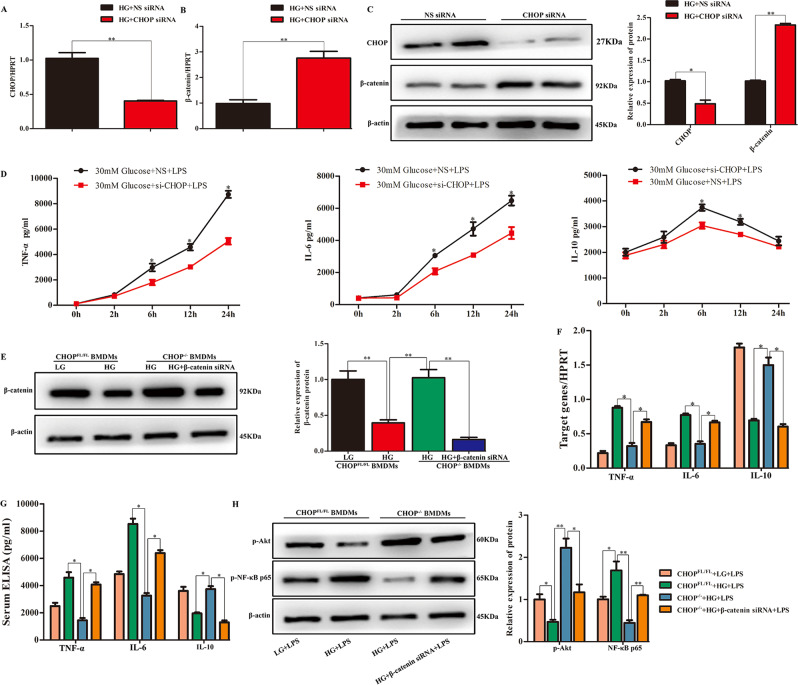
β-Catenin negative regulation by CHOP was critical for HG-mediated inflammatory responses in macrophages. **A**, **C** CHOP in differentiated BMDMs cultured in HG conditions was inhibited by transfection of siRNA, and endogenous CHOP was validated by qRT-PCR and western blot. **P < 0.01. **B**, **C** β-Catenin levels were increased after CHOP downregulation in hyperglycemia-stressed BMDMs, as shown by qRT-PCR and western blot. *P < 0.05, **P < 0.01. **D** TNF-α, IL-6, and IL-10 concentration in culture supernatants were measured by ELISA at 0, 2, 6, 12, and 24 h. **E** Western blot detection of β-catenin expression in different groups of BMDMs with LPS (1 µg/ml) stimulation. **F** Quantification analysis of mRNAs of cytokines TNF-α, IL-6, and IL-10 (6 h). **G** Cytokine in supernatants was evaluated by ELISA (24 h). *P < 0.05. **H** Protein expression of p-Akt, p-NF-κB p65, and β-actin (6 h) was analyzed using western blotting. *P < 0.05, **P < 0.01. Data stand for three independent experiments with similar results.

To further investigate the regulatory role of CHOP on β-catenin under HG conditions, BMDMs cells with deficient CHOP expression were established using siRNA, and the knockdown efficiency of CHOP was verified (Fig. 7A). Inhibition of CHOP enhanced mRNA and protein expression of β-catenin under HG medium treatment (Fig. 7B, C). Functionally, downregulated CHOP affected IL-6, TNF-α, and IL-10 concentration in HG-stressed BMDMs in response to LPS stimulation (Fig. 7D).

To elucidate the function of β-catenin in CHOP-mediated innate immune responses under HG conditions, BMDMs were isolated from WT and CHOP^−/−^ mice and maintained in Dulbecco’s Modified Eagle Medium (DMEM) with 5 mM or 30 mM glucose. HG effectively inhibited β-catenin expression in WT BMDMs, but CHOP deficiency markedly reversed the inhibition of β-catenin expression, which was then silenced by β-catenin siRNA (Fig. 7E). We stimulated these BMDMs with LPS for 6 and 24 h and analyzed cytokine levels, including TNF-α, IL-6, and IL-10, by quantitative reverse transcription–polymerase chain reaction (qRT-PCR; 6 h) and ELISA (24 h). HG-enhanced IL-6 and TNF-α were reduced in CHOP-deficient BMDMs, while HG-inhibited IL-10 was increased after LPS treatment. Notably, β-catenin siRNA almost abolished the anti-inflammatory function of CHOP deficiency in the above conditions (Fig. 7F, G). Moreover, CHOP deficiency restored p-Akt expression, but this effect was neutralized by β-catenin siRNA after LPS treatment in HG-derived BMDMs. The opposite effect was observed in relation to p-NF-κB p65 factor level (Fig. 7H).

### Administration of HG-cultured macrophages exacerbated hepatic IRI by ATF6-CHOP axis mediated β-catenin

**Fig. 8:**
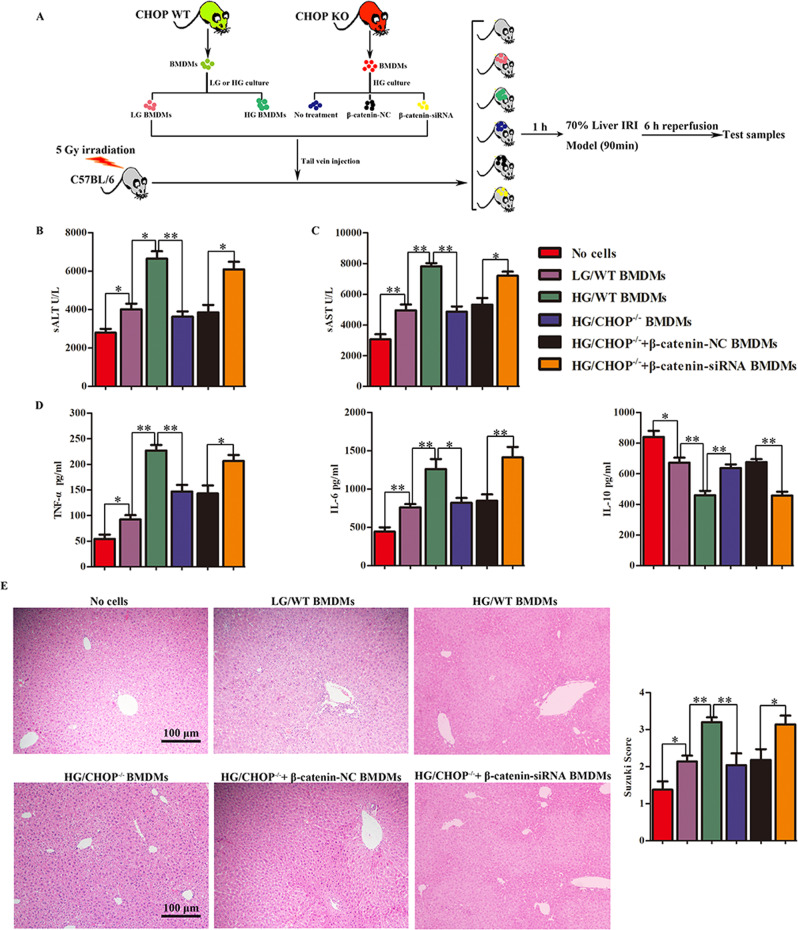
Administration of HG-cultured macrophages exacerbated hepatic IRI by ATF6–CHOP axis-mediated β-catenin. **A** Flow chart of administration of HG-cultured macrophages into mice. **B**, **C** Liver function was evaluated by sALT and sAST level (*n* = 6). *P < 0.05, **P < 0.01. **D** Inflammatory cytokine levels in serum in different groups were determined by ELISA. **E** Histopathologic analysis of livers harvested 6 h after 90 min of liver ischemia, and severity elevation of liver injury was judged according to Suzuki’s histological grading. *P < 0.05, **P < 0.01.

Before receiving the injection of exogenous BMDMs, it is necessary to destroy the native bone marrow of WT mice by irradiation (5 Gy). BMDMs extracted from WT and CHOP^−/−^ mice were maintained in HG or LG condition and induced to differentiate for 7 days. Experimental mice were separated into six groups, including no cells, LG/WT BMDMs, HG/WT BMDMs, HG/CHOP^−/−^ BMDMs, HG/CHOP^−/−^ + β-catenin-NC BMDMs and HG/CHOP^−/−^ + β-catenin-siRNA BMDMs. Then, the CHOP^−/−^ BMDMs maintained under HG DMEM were delivered with siRNAs. Subsequently, before liver IR, the number of 5 × 10^6^ BMDMs was injected into the recipient mice whose bone marrow had been destroyed by tail vein injection. At 6 h post reperfusion and 1 h 30 min post ischemia, we collected liver tissues and serum for analysis (Fig. 8A). The HG/WT BMDMs group had increased sALT and sAST levels compared with the LG/WT BMDMs group. HG/CHOP^−/−^ BMDMs group had significantly decreased level of sALT and sAST compared with WT BMDMs differentiated in HG medium (HG/WT BMDMs), whereas injection of CHOP^−/−^ BMDMs disrupting β-catenin markedly elevated sALT and sAST levels (Fig. 8B, C). Furthermore, TNF-α and IL-6 protein expression was markedly enhanced and serum IL-10 expression level was significantly reduced in mice injected with HG/WT BMDMs compared with the LG/WT BMDMs group. Compared with the HG/WT BMDMs group, the IL-6 and TNF-α in mice injected with HG/CHOP^−/−^BMDMs decreased, but the expression of IL-10 protein increased significantly. Disruption of β-catenin in CHOP^−/−^ BMDMs enhanced the abundance of IL-6 and TNF-α, and suppressed IL-10 in serum compared with the HG/CHOP^−/−^ + β-catenin-NC BMDMs-injection group (Fig. 8D). Compared with the HG/WT BMDMs group, LG/WT BMDMs group showed more minor liver structure damage. The HG/CHOP^−/−^ BMDMs group had relieved liver tissue architecture damage, and its Suzuki scores were lower than that of HG/WT BMDMs. Administration of CHOP^−/−^ BMDMs with deficient β-catenin expression partially reversed this influence (Fig. 8E).

## Discussion

The present study first provides evidence for the role and mechanisms of hyperglycemia in triggering ER stress, and its effect on inflammatory responses and liver injury during IR. The current study demonstrated that hyperglycemia specifically activated the ATF6-CHOP pathway of ER stress in liver tissues and KCs in DM patients and hyperglycemic mice. Furthermore, this pathway was crucial for hyperglycemia-exacerbated inflammatory responses, hepatocellular apoptosis/necrosis, and liver injury after IR. The hyperglycemia-triggered ATF6-CHOP pathway was also shown to regulate innate immune responses, and NF-κB and Akt activity in a β-catenin-dependent manner.

Epidemiology and a series of clinical data show that when diabetic patients develop IRI, the patient’s complications are more serious. Moreover, diabetes and hyperglycemia are associated with multiple ischemia tissue injuries. Research on the lung showed that acute hyperglycemic exacerbation of lung IRI was mediated by the receptor for advanced glycation end-products signaling [25]. DM disrupted the cardiac gene expression profile in the heart, with possible implications for the development of cardiac complications [5]. Another study revealed that peripheral nerve regeneration in DM mice with acute nerve injury is hindered. The recovery of this process requires macrophages to participate in the mediation, and the coordination of inflammation and tissue repair [26]. The liver is an important organ of glucose metabolism, and patients with liver disease are therefore prone to hyperglycemia, and approximately 25% of patients with liver surgery experience diabetes. Type I diabetes can be used as a predictive factor for prognostic in individuals undergoing liver transplantation and liver resection [27]. The effect of hyperglycemia on hepatic injury is thus of particular importance. Wang et al. found that HG significantly worsened the pathology of liver fibrosis, while HG stimulated extracellular matrix production and proliferation of HSCs and promoted the effect of PDGF-BB on their bio-function at the cellular level [28]. In light of these findings, Zhang et al. demonstrated that hyperglycemia could aggravate liver IRI, which was related to oxidative stress and inflammation [29]. However, the exact mechanisms whereby hyperglycemia aggravated liver IRI remain unclear.

IR is accompanied by oxidation, lack of energy, and intracellular Ca^2+^ imbalance, which are factors that can lead to ER stress. In the heart, attenuation of ER stress by nesfatin-1 inhibited myocardial IRI, and in contrast, induction of ER stress by tunicamycin (TM) abrogated nesfatin-1-mediated protection against myocardial IRI [30]. A previous investigation demonstrated that ER stress in the brain induced by TM protected against transient ischemic brain injury, and suggested that PARK2-mediated mitophagy might underlie the protection of ER stress [31]. Accumulating studies have reported the involvement of ER stress in liver IRI. PBA, an inhibitor of ER stress, protected against liver IRI by inhibiting ER-stress-mediated apoptosis [32]. Indeed, IR-activated ER stress was characterized by signaling specificity, cell specificity, and time-dependence in the ischemic liver. Chevet et al. demonstrated that distinct ER stress was triggered during each phase of liver transplantation [33]. We previously showed that warm ischemia specifically activated ATF6 in KCs, and then promoted NF-κB activation and inhibited Akt activation upon TLR stimulation, which induced serious pro-inflammatory response [34]. Yang et al. reported that hepatocyte-specific mesencephalic astrocyte-derived neurotrophic factor knockout activated ATF4-CHOP pathway, ultimately aggravating hepatic IRI [35]. However, whether hyperglycemia specifically activates KCs ER stress signaling pathways and exacerbates acute inflammatory hepatic injury remains unclear.

The molecular mechanisms of IR-activated TLR4-related innate immunity may involve multiple types of signaling pathways. β-catenin is critical for modulating the innate immune response in liver injury by regulating its Akt-mediated signaling [36]. Akt signaling activation elevates β-catenin activity and represses the TLR4-mediated innate immune response during liver IRI [37]. Depression of the hepatocyte E-cadherin/β-catenin interaction mediated by tissue inhibitor of metalloproteinase 3 deficiency and knockdown of β-catenin expression induced caspase activation, and subsequently enhanced apoptosis after hepatic IRI [38]. A recent study demonstrated that myeloid-specific Foxo1-deficient promoted β-catenin-mediated Gli1/Snail signaling activation, and alleviated oxidative stress-induced hepatic injury with decreased accumulation of macrophage/neutrophil in liver IRI. Mechanism analysis showed that Foxo1 directly bounded to endogenous β-catenin in the nucleus, and Foxo1–β-catenin complex in macrophages reduced β-catenin–TCF4 binding via LPS [39]. Then, we investigated the relationship between the ATF6–CHOP axis and β-catenin signaling in the modulation of inflammation. The results showed that hyperglycemia-triggered ATF6-CHOP inhibited β-catenin signaling, and CHOP deficiency accordingly effectively restored hyperglycemia-inhibited β-catenin expression. β-catenin knockdown restored hyperglycemia-related inflammatory responses and hepatic IRI in CHOP^−/−^ mice in vivo, thus implicating β-catenin as a key regulator of innate immunity in hyperglycemia-triggered ATF6-CHOP promotion of inflammation.

In conclusion, this study systemically revealed that diabetes-associated hyperglycemia markedly deteriorated liver IR damage via activation of the ER stress-ATF6-CHOP signaling pathway, which subsequently inhibited β-catenin signaling and promoted TLR4-mediated inflammatory responses (Supporting Fig. S3). Our findings provide the rationale for a potential therapeutic candidate for ameliorating hyperglycemia-exacerbated liver IRI.

## Materials and methods

### Study subjects

Clinical liver samples were surgically taken from 15 enrolled individuals who were diagnosed with benign liver disease and DM. Another 15 healthy subjects were recruited as controls. The clinical characteristics of enrolled patients and controls are presented in Table S1. The informed consent of all recruited individuals was obtained, and the research protocol was approved by the local ethics committee of Nanjing Medical University.

### Animals

WT C57BL/6 male mice and male C57BL/6 CHOP^−/−^ mice (8 weeks) were brought from the Model Animal Research Center of Nanjing University. Genotyping for the experimental mice was carried out using PCR [40]. All animals were raised according to guidance for the Care and Use of Laboratory Animals formulated by the Ministry of Science and Technology of the People′s Republic of China. Protocols for mice experiments were approved by the Institutional Animal Care and Use Committee (IACUC) of Nanjing Medical University (Protocol Number IACUC-1702001).

### Establishment of diabetes and liver IRI models

Randomly separate groups of mice were intraperitoneally injected with STZ or sodium citrate buffer (vehicle control) at 40 mg/kg for 5 days, followed by blood glucose measurement in the tail vein at day 14. If the blood glucose concentration of the mice is over 300 mg/dl, these mice are considered diabetic.

The atraumatic clip was adopted to block the blood flow from portal venous and arterial to the left lobe and mid-hepatic lobe for 90 min according to the previous research [41]. The control group was subjected to the same operations except for the closure of blood vessels. The PBA acts as a chemical chaperone and help to restore the proper conformation of unfolded proteins, whose accumulation activates ER stress. PBA (Sigma, St. Louis, MO, USA) was administered at 100 mg/kg, intraperitoneally, 1 h prior to induction of ischemia. At 0, 6, and 24 h post reperfusion, mice were sacrificed to harvest liver and serum samples.

### Serum biochemical examination

Serum concentrations of ALT (sALT) and AST (sAST) were quantified by Olympus AU5400 chemistry analyzer (Olympus, Tokyo, Japan).

### Histology and immunohistochemistry (IHC)

The collected liver specimens were fixed with 10% formaldehyde, and 4μm thick paraffin sections were made and stained with hematoxylin and eosin (HE). The severity of liver IRI was graded in a blinded manner using Suzuki’s criteria, ranging from 0 to 4 [42]. CD68+ macrophages and Ly6G+ neutrophils in collected liver samples were detected by IHC analysis [43].

### IF staining

IF staining of CHOP was performed on liver tissues from DM patients and healthy subjects. Liver samples from sham-treated control and diabetic mice, and IR model were fixed in 4% neutral formaldehyde, embedded in Tissue Tek OCT compound (Electron Microscopy Sciences, Japan), and 6-µm frozen sections were made for IF analysis. To reduce background interference, the sections were blocked with 3% bovine serum albumin (BSA), then reacted with rat anti-CD68 monoclonal antibody (mAb, 1:100, Abcam, Cambridge, UK), anti-CHOP mouse mAb (1:100, Cell Signaling Technology, Danvers, MA, USA) and Cy3- conjugated secondary antibody (1:100, Jackson Immuno-Research, West Grove, PA, USA). Mounting solution mixed with DAPI dye and anti-fluorescence quencher was added on the surface of sections for confocal microscope (LSM510, Carl Zeiss MicroImaging GmbH, Jena, Germany) detection.

### Cleaved caspase-3 and TUNEL staining

Cleaved caspase-3 in liver tissues was measured by immunohistological staining with anti-cleaved caspase-3 (Cell Signaling Technology, Danvers, MA, USA) according to the commercial manual. The sample sections were detected via TUNEL using in situ cell death detection kit (Roche-Boehringer, Mannheim, Germany).

### Quantitative RT-PCR

Total RNA was extracted using TRIzol reagent (Invitrogen, Carlsbad, CA, USA), and the first line of cDNA was synthesized by Primescript RT reagent (Takara, Shiga, Japan). Quantitative PCR (qPCR) was carried out using a 7900 Real-Time PCR System (Applied Biosystems, Foster City, CA, USA) through Fast Start Universal SYBR Green Master (Takara, Japan). Levels of mRNA were quantitated by the comparative CT method, and the target gene was normalized to HPRT or β-actin. Sequences of primers used in qRT-PCR were summarized in Table S2.

### Western blot

The concentration of the protein sample was examined using a BCA protein assay kit (Beyotime, Shanghai, China). Different molecules were separated by SDS/PAGE (10%) and were transferred into PVDF membranes (Bio-Rad, Hercules, CA, USA). Antibodies against cATF6 (Abcam, Cambridge, UK), CHOP, β-catenin, Bcl-xL, Bcl-2, Cleaved caspase-3, Bax, p-Akt, p-NF-κB p65, p-IKBα, p-IKKα/β, GAPDH, β-actin (Cell Signaling Technology, Danvers, MA, USA) were added to incubate membranes at 4 °C overnight. Secondary antibodies conjugated with HRP (Cell Signaling Technology) were then applied to react with the membrane at room temperature for 1 h. Finally, SuperSignal West Pico Chemiluminescent Substrates (Thermo Fisher Scientific, Rockford, IL, USA) were dropped onto the PVDF membrane to enhance the detection signal of the protein bands.

### Knockdown of β-catenin via siRNA delivery

The small interfering RNA (siRNA) sequences for mouse β-catenin were as follows: 5′-CCAGGUGGUAGUUAAUAAATT-3′; nonspecific (NS): 5′-CGAATCCACAAAGCGCGCTT-3′. For cellular analysis, BMDMs were transfected in vitro with siRNA using Lipofectamine 3000 (Invitrogen, San Diego, CA, USA). For animal assays, nonspecific (NS) siRNA and β-catenin siRNA (2 mg/kg) were labeled with Alexa Fluor 488 and were respectively mixed with mannose-conjugated polymers (Polyplus transfection^TM^, Illkirch, France), followed by injection into mice through tail vein at 4 h prior to liver ischemia.

### KC culture

Mice livers were perfused in situ via the superior mesenteric vein with phosphate-buffered saline and were digested using 0.27% collagenase IV (Sigma, Saint Louis, MO, USA) at 37 °C for 40 min. Perfused livers were filtered through a cell strainer (70 µm) to remove undigested tissue, and the collected filtrate was transferred into a 50 ml tube and centrifuged at 250 × *g* for 5 min at 4 °C to pellet the cells. Then the isolated cells were suspended in 30 ml DMEM containing 10% fetal bovine serum (FBS), and non-parenchymal (NPCs) were separated from parenchymal cells by differential centrifugation. The isolated NPCs were seeded in six-well cell culture plates filled with 1.5 ml DMEM supplemented with 10% FBS, 10 mM HEPES, 2 mM GlutaMax, 100 U/ml penicillin, and 100 mg/ml streptomycin. After being cultured in a cell incubator at 37 °C for 1 h, the non-adherent cells were removed by refreshing the medium. The left KC cells were cultured in an atmosphere of 5% CO_2_ at 37 °C for further in vitro laboratory studies.

### BMDM isolation and differentiation

BMDMs were obtained from the bone marrow of 6~10 weeks-old mice and cultured in 5 mM (low) or 30 mM (high) glucose DMEM with 10% FBS, 20% L929 conditioned medium, 100 U/ml penicillin, and 100 mg/ml streptomycin for 6 days. At the 7-day post cell seeding, differentiated BMDMs were plated under the same glucose conditions. The cell purity was determined as 94%~99% F4/80+. Lipopolysaccharide (LPS) (1 µg/ml; InvivoGen, San Diego, CA, USA) was added for 0–24 h treatment to initiate the differentiation. The cell supernatants were removed to new tubes at indicated time points for cytokines measurement.

### In vitro siRNA transfection

The siRNA sequence (5′-GCUAGCUGAAGAGAACGAGTT-3′) targeting mouse CHOP gene and NS control sequence (5′-UUCUCCGAACGUGUCACGUTT-3′) were entrusted to GenePharma for chemical synthesis (Shanghai, China). BMDMs differentiated under high-glucose conditions were transfected with 30 nmol/ml siRNA using Lipofectamine 3000 reagent (Invitrogen, CA, USA) following commercial guidance. At 6 h post transfection, the medium was replaced with fresh DMEM and the cells were treated with LPS for a further 0~24 h.

### Enzyme-linked immunosorbent assay

Cytokines in supernatants or serum were quantified according to the commercial protocols (eBioscience, San Diego, CA, USA). The measurement values were obtained and recorded using a Multiscan FC plate reader (Thermo Scientific, MA, USA).

### Construction of chimeric mice

A cesium source at 5 Gy was employed to conduct the irradiation for mice. Before irradiation, isoflurane was used to anesthetize mice. After being irradiated, the mice are divided into cages and reared in accordance with normal conditions. Before injection, Mouse BMDMs were differentiated in 5 mM or 30 mM glucose DMEM for a week and were transfected with siRNAs after 7 days of culture. Subsequently, BMDMs at a density of 5 × 10^6^ were injected into each recipient myeloid-destructive mice through tail vein injection before liver IR in the respective groups. The mice were sacrificed at 6 h after reperfusion for further experiments.

### Statistical analysis

All data are expressed as mean ± standard deviation (SD) based on at least three independent experiments. One-way analysis of variance tests was used for significant difference analysis among three groups, and Mann–Whitney *U*-tests were used for comparisons between two groups. All the *P* values were two-sided, and *P* < 0.05 indicated statistically significant.

## Supplementary information

Supplementary information

Figure S1

Figure S2

Figure S3

Original western blots

Table S1

Table S2

## Data Availability

All data supporting the findings of this study are available within the paper.
