# Supplementary material for: Hyperglycemia-triggered ATF6-CHOP pathway aggravates acute inflammatory liver injury by β-catenin signaling
Source: Cell Death Discov. 2022 Mar 14;8:115. doi: 10.1038/s41420-022-00910-z (PMC8921205; doi:10.1038/s41420-022-00910-z)
Supplement: Supplementary file 1 — Supplementary information [file 41420_2022_910_MOESM1_ESM.docx]

**Supplementary figure legends**

**Supporting Fig. S1 Mice blood glucose levels and cleaved caspase-3 expression in liver tissues.** Diabetic (STZ) and control mice were prepared as described in the Materials and Methods. **(A)** Blood glucose levels were measured at 14 days post-first STZ injection prior to the start of liver ischemia experiments. Representative of 6 mice/group. ***P<0.001. **(B and C)** Cleaved caspase-3 staining of liver sections (×200). Cleaved caspase3+ cells were quantitated by counting the numbers of positive cells/area. Mean ± SD (n=4-6 samples/group). *P<0.05. Scale bars, 50 μm.

**Supporting Fig. S2 Hyperglycemia-triggered ATF6-CHOP pathway depressed β-catenin signaling in liver tissues.** **(A)** qRT-PCR-assisted detection of β-catenin mRNA in control mice, diabetic mice, and PBA-treated diabetic mice after either sham operation or 90 min of liver ischemia and 6 h of reperfusion. Each column represents the mean ± SD (n=3-4 mice/group). *P<0.05, **P<0.01. **(B)** qRT-PCR-assisted detection of β-catenin in WT control, CHOP^−/−^ and WT diabetic mouse ischemic livers. Mean ± SD (n=3-4 samples/group). **P<0.01.

**Supporting Fig. S3 Schematic representation of signaling pathway by which hyperglycemia specifically triggers ER stress-ATF6-CHOP signaling, inhibits β-catenin activity, promotes inflammatory responses, and exacerbates liver IRI.**

**Supporting Fig. S4 Uncropped images of blots presented in main Figure 1.**

**Supporting Fig. S5 Uncropped images of blots presented in main Figure 2.**

**Supporting Fig. S6 Uncropped images of blots presented in main Figure 3E.**

**Supporting Fig. S7 Uncropped images of blots presented in main Figure 4D.**

**Supporting Fig. S8 Uncropped images of blots presented in main Figure 5.**

**Supporting Fig. S9 Uncropped images of blots presented in main Figure 6.**

**Supporting Fig. S10 Uncropped images of blots presented in main Figure 7.**

**Supplementary table legends**

**Table S1** **Patient characteristics.**

**Table S2 Information of primers.**
