# Supplementary figures and images for: Hyperglycemia-triggered ATF6-CHOP pathway aggravates acute inflammatory liver injury by β-catenin signaling
Source: Cell Death Discov. 2022 Mar 14;8:115. doi: 10.1038/s41420-022-00910-z (PMC8921205; doi:10.1038/s41420-022-00910-z)

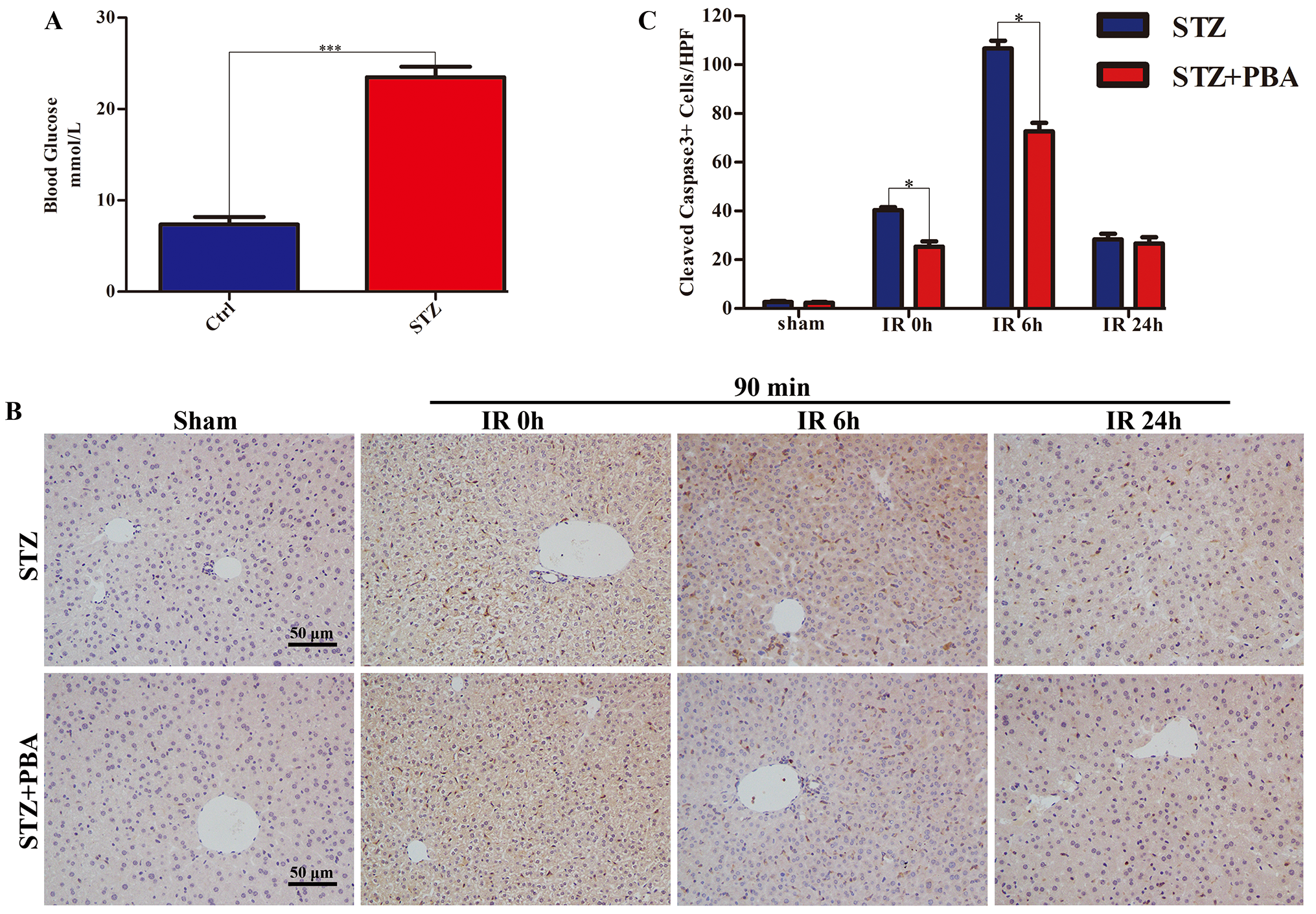

Supplement: Supplementary file 2 — Figure S1 [file 41420_2022_910_MOESM2_ESM.tif]

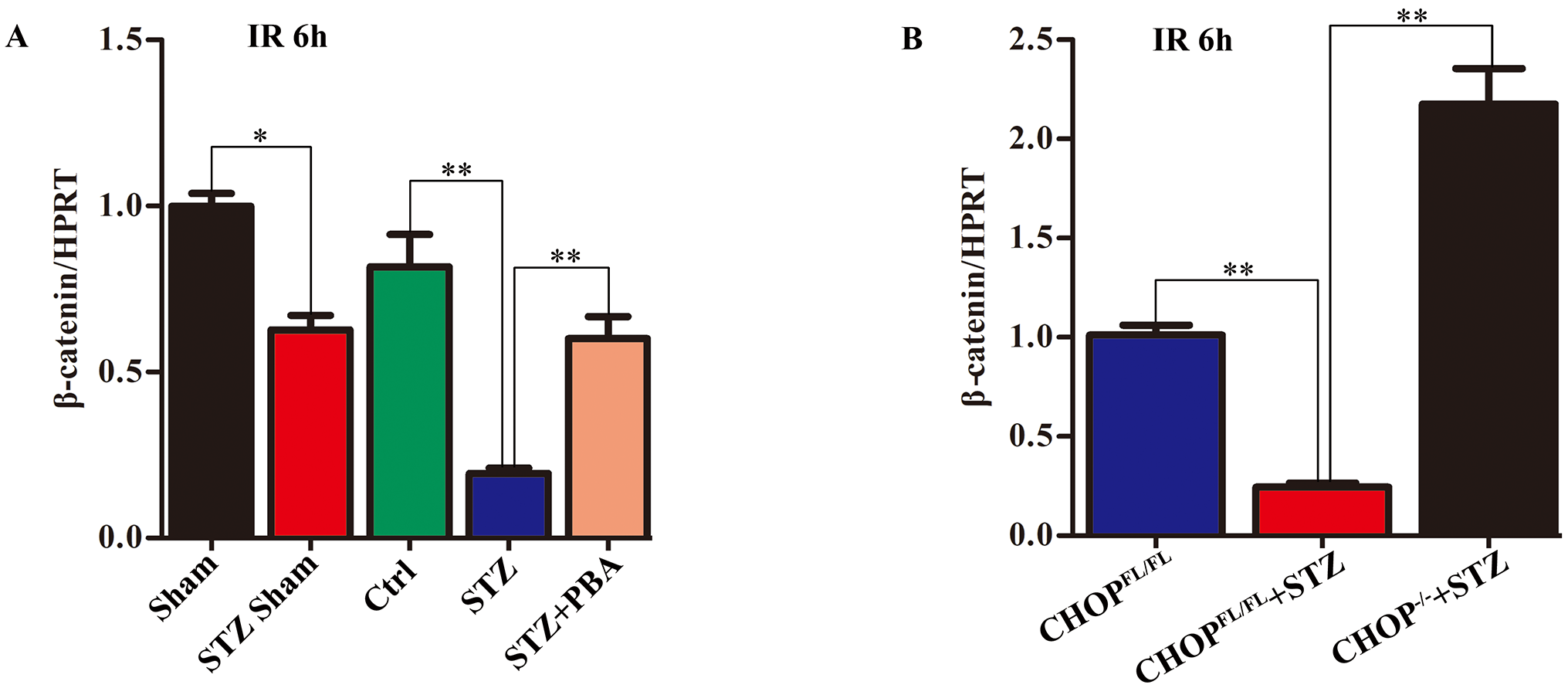

Supplement: Supplementary file 3 — Figure S2 [file 41420_2022_910_MOESM3_ESM.tif]

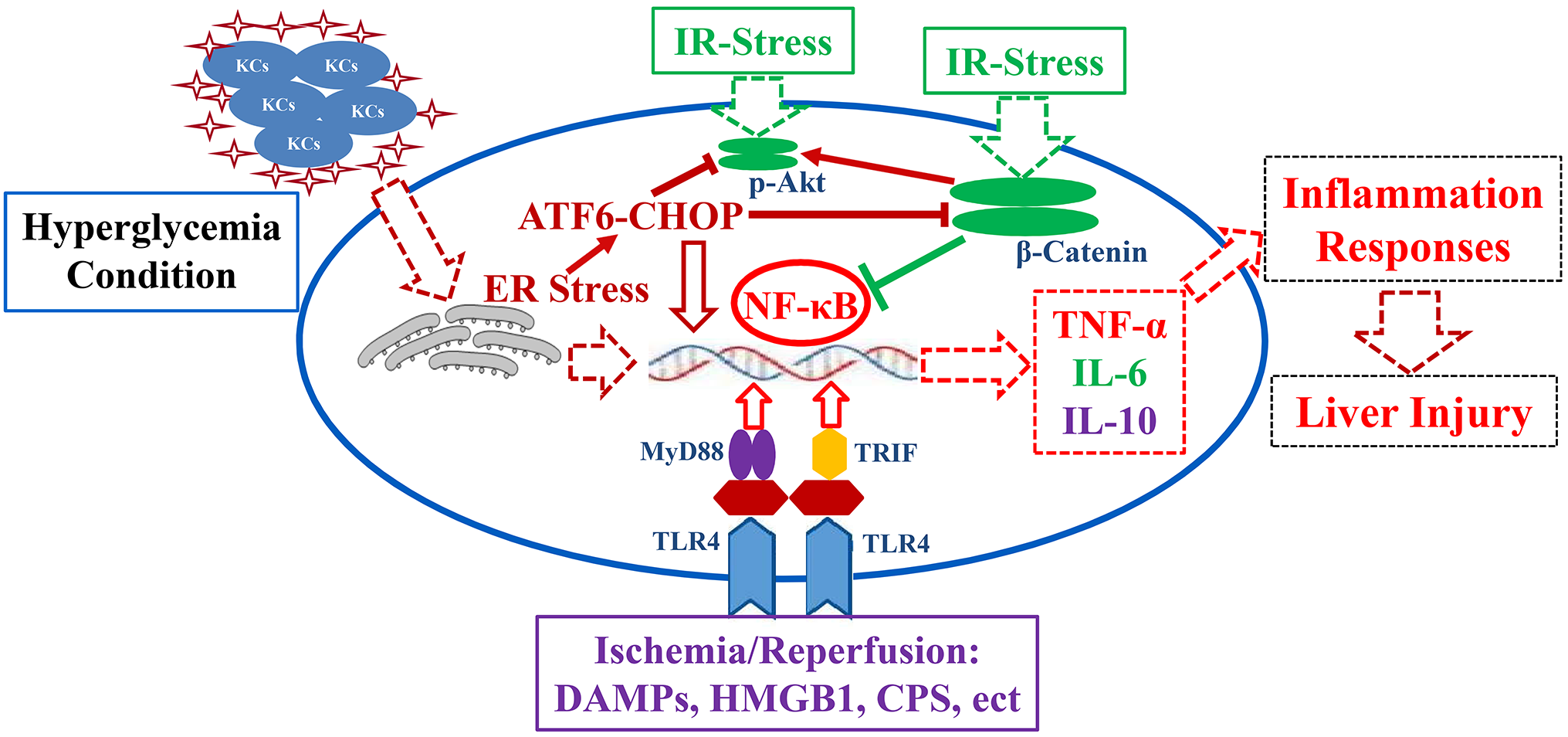

Supplement: Supplementary file 4 — Figure S3 [file 41420_2022_910_MOESM4_ESM.tif]
