# Supplementary material for: Hyperglycemia-triggered ATF6-CHOP pathway aggravates acute inflammatory liver injury by β-catenin signaling
Source: Cell Death Discov. 2022 Mar 14;8:115. doi: 10.1038/s41420-022-00910-z (PMC8921205; doi:10.1038/s41420-022-00910-z)
Supplement: Supplementary file 5 — Original western blots [file 41420_2022_910_MOESM5_ESM.pdf]

**Figure S4**

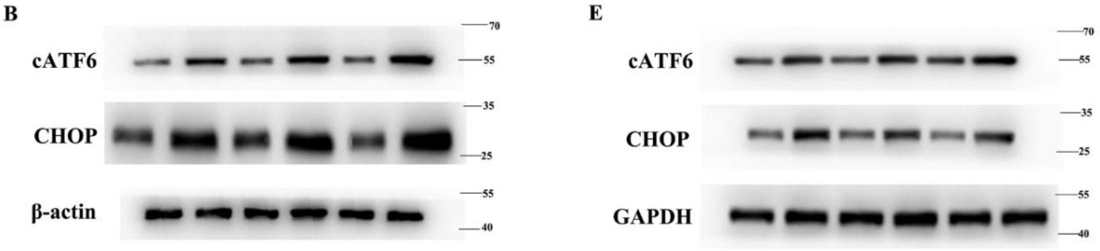


**Uncropped images of blots presented in main Figure 1.**

**Figure S5**

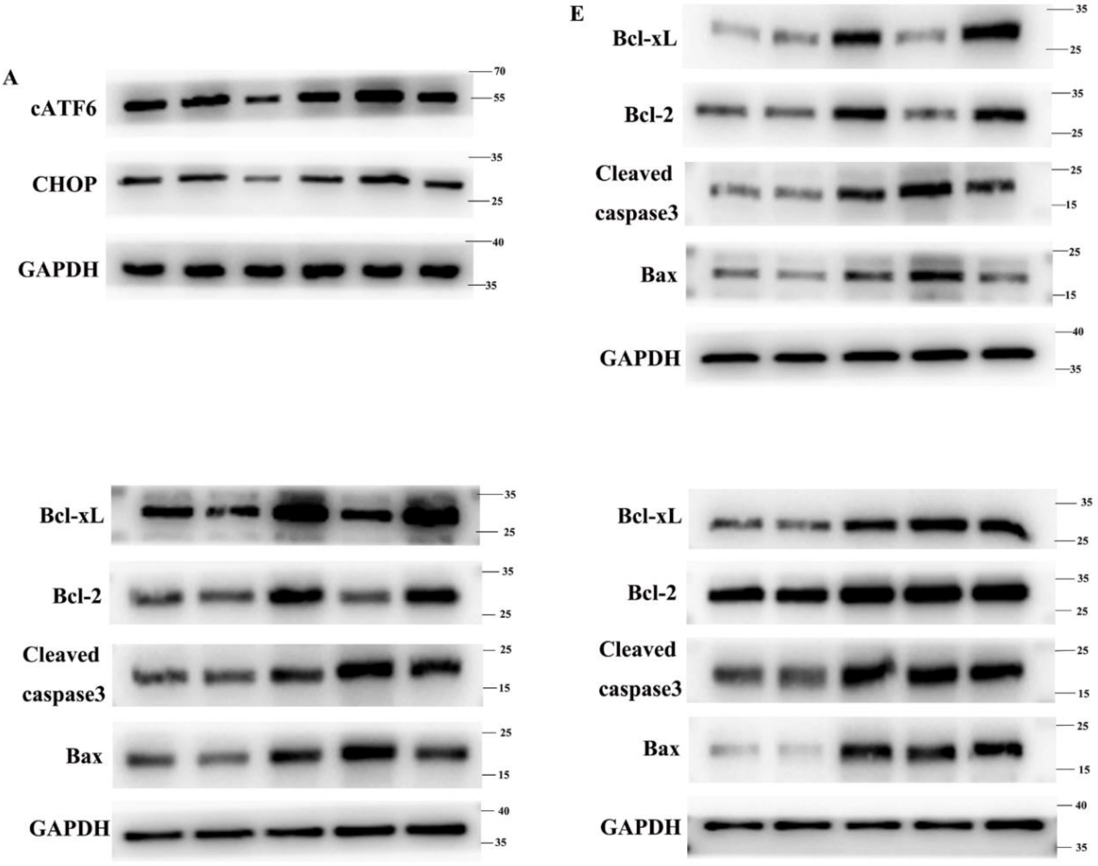


**Uncropped images of blots presented in main Figure 2**

**Figure S6**

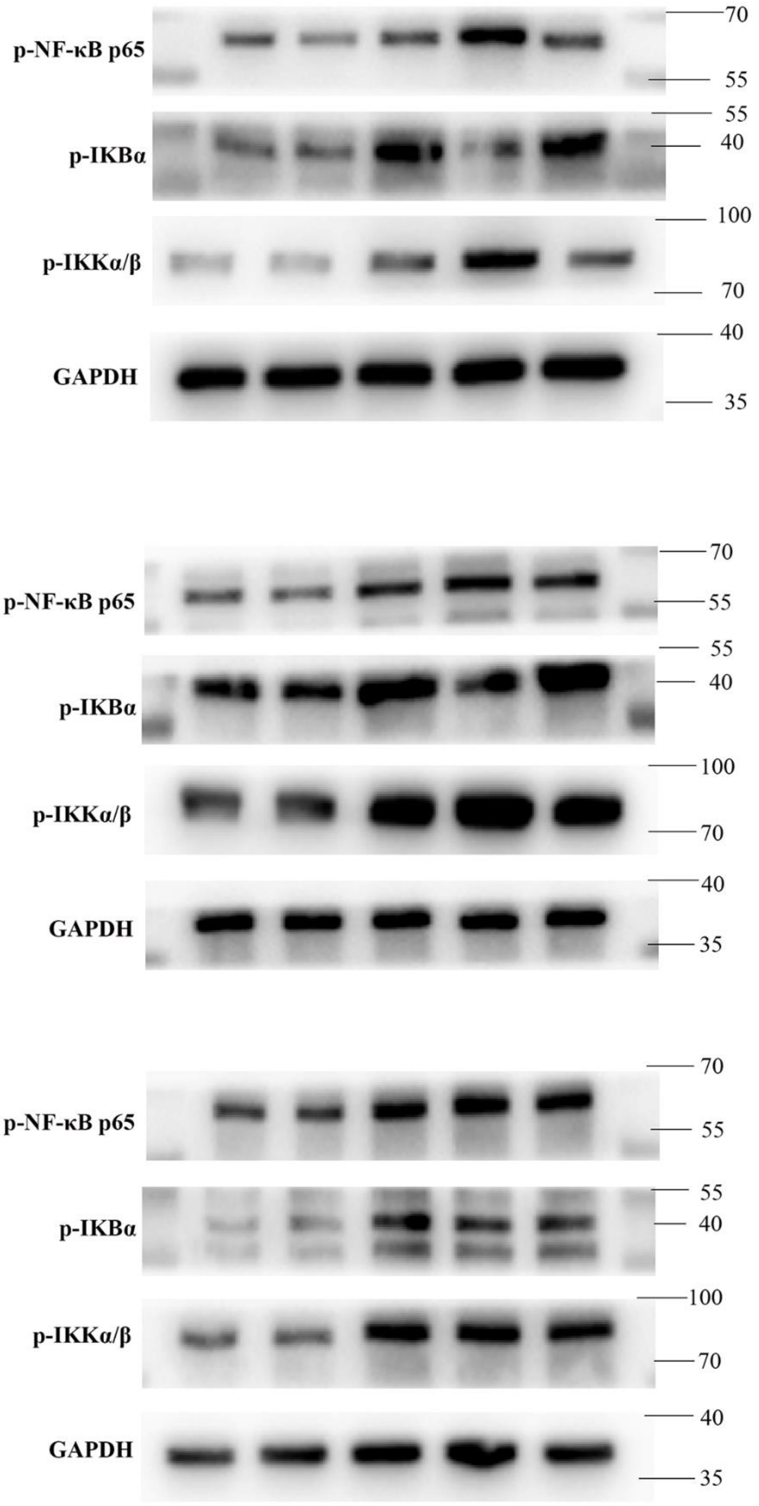


**Uncropped images of blots presented in main Figure 3E.**

**Figure S7**

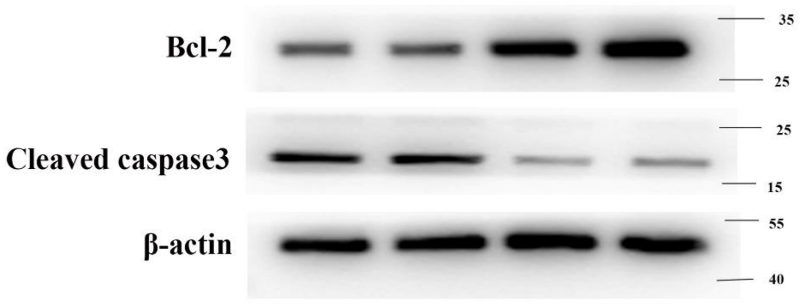


**Uncropped images of blots presented in main Figure 4D.**

**Figure S8**

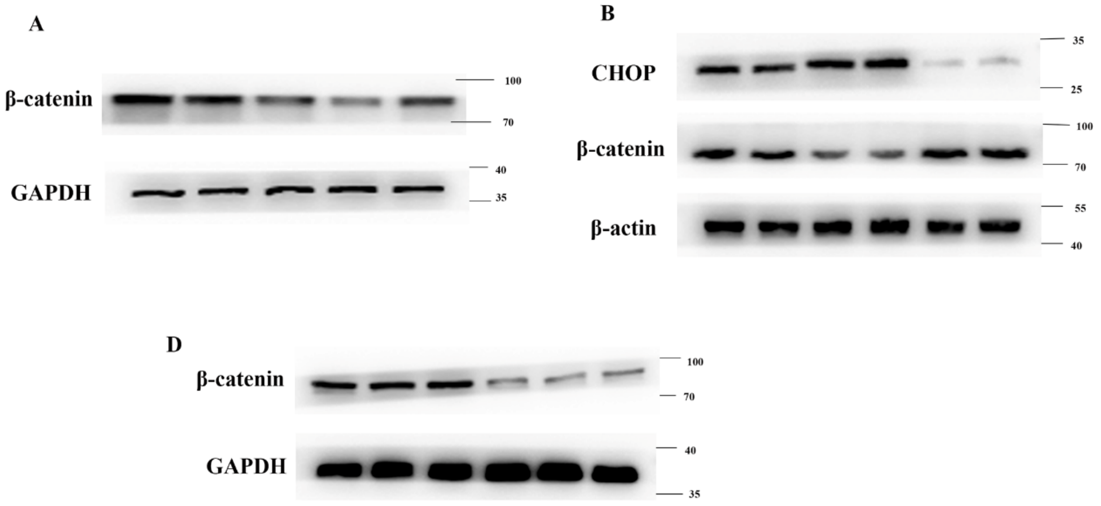


**Uncropped images of blots presented in main Figure 5.**

**Figure S9**

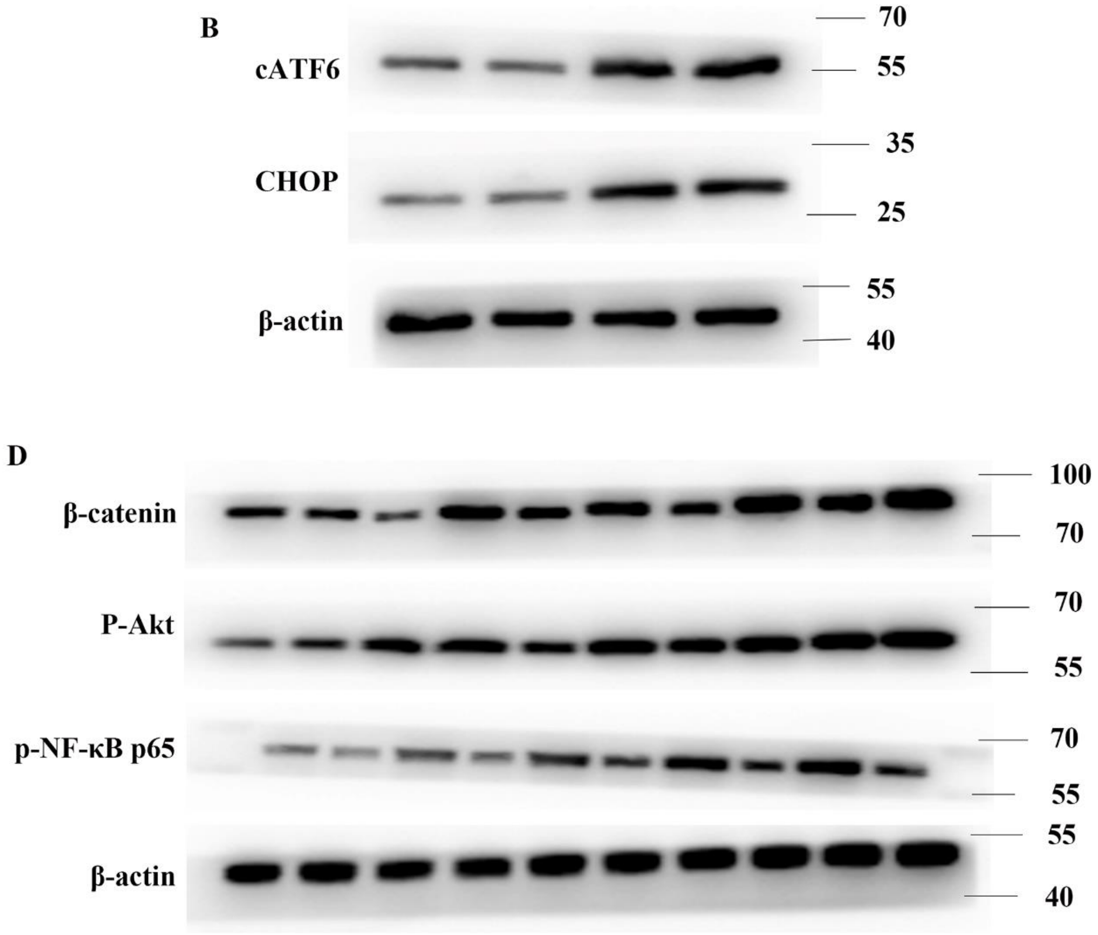


**Uncropped images of blots presented in main Figure 6.**

**Figure S10**

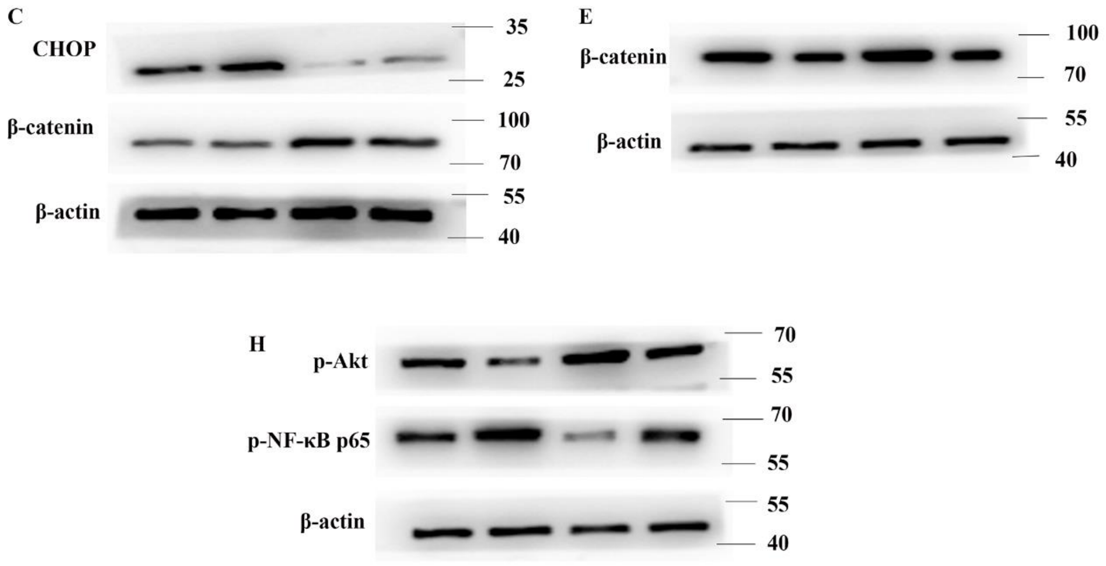


**Uncropped images of blots presented in main Figure 7.**
