# Supplementary material for: Hyperglycemia-triggered ATF6-CHOP pathway aggravates acute inflammatory liver injury by β-catenin signaling
Source: Cell Death Discov. 2022 Mar 14;8:115. doi: 10.1038/s41420-022-00910-z (PMC8921205; doi:10.1038/s41420-022-00910-z)
Supplement: Supplementary file 6 — Table S1 [file 41420_2022_910_MOESM6_ESM.docx]

**Table S1** **Patient characteristics**

| Variables | DM | Ctrl | *p*-value |
| --- | --- | --- | --- |
| N | 15 | 15 |  |
| Sex (male/female) | 8/7 | 9/6 | 0.713 |
| Age (year) | 62.6 ± 7.85 | 60.7 ± 7.71 | 0.517 |
| Diseases |  |  |  |
| Hepatic hemangioma | 7 | 6 |  |
| Hepatic cyst | 6 | 8 |  |
| FNH | 2 | 1 | 0.706 |
| ALT |  |  |  |
| POD1 | 375.8 ± 45.19 | 268.9 ± 38.38 | ＜0.001^***^ |
| POD3 | 252.8 ± 53.84 | 152.3 ± 26.09 | ＜0.001^***^ |
| POD5 | 79.5 ± 14.24 | 74.4 ± 22.11 | 0.461 |

Abbreviation: DM, Diabetes Mellitus; FNH, Focal Nodular Hyperplasia; POD, Post Operation Day.

All data are presented as the mean ± SD. ^*^*p* <0.05, ^**^*p* <0.01, ^***^*p* <0.001.
