# Supplementary material for: Hyperglycemia-triggered ATF6-CHOP pathway aggravates acute inflammatory liver injury by β-catenin signaling
Source: Cell Death Discov. 2022 Mar 14;8:115. doi: 10.1038/s41420-022-00910-z (PMC8921205; doi:10.1038/s41420-022-00910-z)
Supplement: Supplementary file 7 — Table S2 [file 41420_2022_910_MOESM7_ESM.docx]

**Table S2 Information of primers**

| Sequences | Forward (5′ to 3′) | Reverse (5′ to 3′) |
| --- | --- | --- |
| Human-IRE1 | CACAGTGACGCTTCCTGAAAC | GCCATCATTAGGATCTGGGAGA |
| Human-XBP1 | CCCTCCAGAACATCTCCCCAT | ACATGACTGGGTCCAAGTTGT |
| Human-PERK | GGAAACGAGAGCCGGATTTATT | ACTATGTCCATTATGGCAGCTTC |
| Human-ATF4 | ATGACCGAAATGAGCTTCCTG | GCTGGAGAACCCATGAGGT |
| Human-CHOP | GGAAACAGAGTGGTCATTCCC | CTGCTTGAGCCGTTCATTCTC |
| Human-ATF6 | AGCAGCACCCAAGACTCAAAC | GCATAAGCGTTGGTACTGTCTGA |
| Mouse-IRE1 | GTGGTCTCCTCTCGGGTTC | CCGTCCCAGGTAGACACAAAC |
| Mouse-XBP1 | AGCAGCAAGTGGTGGATTTG | GAGTTTTCTCCCGTAAAAGCTGA |
| Mouse-PERK | AGTCCCTGCTCGAATCTTCCT | TCCCAAGGCAGAACAGATATACC |
| Mouse-ATF4 | AAGGAGGAAGACACTCCCTCT | CAGGTGGGTCATAAGGTTTGG |
| Mouse-CHOP | CTGGAAGCCTGGTATGAGGAT | CAGGGTCAAGAGTAGTGAAGGT |
| Mouse-ATF6 | TCGCCTTTTAGTCCGGTTCTT | GGCTCCATAGGTCTGACTCC |
| Mouse-β-catenin | ATGGAGCCGGACAGAAAAGC | TGGGAGGTGTCAACATCTTCTT |
| TNF-α | CAGGCGGTGCCTATGTCTC | CGATCACCCCGAAGTTCAGTAG |
| IL-6 | TAGTCCTTCCTACCCCAATTTCC | TTGGTCCTTAGCCACTCCTTC |
| IL-10 | CTTACTGACTGGCATGAGGATCA | GCAGCTCTAGGAGCATGTGG |
| HPRT | TCAACGGGGGACATAAAAGT | TGCATTGTTTTACCAGTGTCAA |
| β-actin | TGACGTGGACATCCGCAAAG | CTGGAAGGTGGACAGCGAGG |
